# Expression profiles of long non-coding RNAs located in autoimmune disease-associated regions reveal immune cell-type specificity

**DOI:** 10.1186/s13073-014-0088-0

**Published:** 2014-10-28

**Authors:** Barbara Hrdlickova, Vinod Kumar, Kartiek Kanduri, Daria V Zhernakova, Subhash Tripathi, Juha Karjalainen, Riikka J Lund, Yang Li, Ubaid Ullah, Rutger Modderman, Wayel Abdulahad, Harri Lähdesmäki, Lude Franke, Riitta Lahesmaa, Cisca Wijmenga, Sebo Withoff

**Affiliations:** Department of Genetics, University of Groningen, University Medical Center Groningen, Groningen, the Netherlands; Turku Center for Biotechnology, University of Turku, and Åbo Akademi University, Turku, Finland; Department of Rheumatology and Clinical Immunology, University of Groningen, University Medical Center Groningen, Groningen, the Netherlands; Department of Information and Computer Science, Aalto University, Espoo, Finland

## Abstract

**Background:**

Although genome-wide association studies (GWAS) have identified hundreds of variants associated with a risk for autoimmune and immune-related disorders (AID), our understanding of the disease mechanisms is still limited. In particular, more than 90% of the risk variants lie in non-coding regions, and almost 10% of these map to long non-coding RNA transcripts (lncRNAs). lncRNAs are known to show more cell-type specificity than protein-coding genes.

**Methods:**

We aimed to characterize lncRNAs and protein-coding genes located in loci associated with nine AIDs which have been well-defined by Immunochip analysis and by transcriptome analysis across seven populations of peripheral blood leukocytes (granulocytes, monocytes, natural killer (NK) cells, B cells, memory T cells, naive CD4^+^ and naive CD8^+^ T cells) and four populations of cord blood-derived T-helper cells (precursor, primary, and polarized (Th1, Th2) T-helper cells).

**Results:**

We show that lncRNAs mapping to loci shared between AID are significantly enriched in immune cell types compared to lncRNAs from the whole genome (α <0.005). We were not able to prioritize single cell types relevant for specific diseases, but we observed five different cell types enriched (α <0.005) in five AID (NK cells for inflammatory bowel disease, juvenile idiopathic arthritis, primary biliary cirrhosis, and psoriasis; memory T and CD8^+^ T cells in juvenile idiopathic arthritis, primary biliary cirrhosis, psoriasis, and rheumatoid arthritis; Th0 and Th2 cells for inflammatory bowel disease, juvenile idiopathic arthritis, primary biliary cirrhosis, psoriasis, and rheumatoid arthritis). Furthermore, we show that co-expression analyses of lncRNAs and protein-coding genes can predict the signaling pathways in which these AID-associated lncRNAs are involved.

**Conclusions:**

The observed enrichment of lncRNA transcripts in AID loci implies lncRNAs play an important role in AID etiology and suggests that lncRNA genes should be studied in more detail to interpret GWAS findings correctly. The co-expression results strongly support a model in which the lncRNA and protein-coding genes function together in the same pathways.

**Electronic supplementary material:**

The online version of this article (doi:10.1186/s13073-014-0088-0) contains supplementary material, which is available to authorized users.

## Background

Autoimmune and immune-related disorders (AID) are a heterogeneous group of disorders that occur in 7 to 9% of people worldwide [1]. These diseases are caused by an inappropriate response of the human immune system against self-antigens. As we have gained more insight into the biological mechanisms underlying different AID, it has become clear that clinically distinct AID with diverse phenotypic manifestations (systemic or organ-specific) share features such as pathophysiological mechanisms, the involvement of human leukocyte antigen (HLA) susceptibility alleles, the production of antibodies to self-antigens, and genetic susceptibility [2-6].

Thus far, many different AID loci have been identified by genome-wide association studies (GWAS) and these are listed in the GWAS catalog [7]. The 186 AID loci known in 2010 resulted in the design of a dedicated SNP array, Immunochip, to fine-map them [8]. By integrating GWAS and Immunochip data with Gencode data from the Encyclopedia of DNA Elements (ENCODE) project, it has become clear that more than 90% of the AID-associated SNPs map to non-coding, regulatory regions [9,10] that may encompass non-coding RNA genes [11]. Using expression quantitative trait loci (eQTLs) analysis, we recently demonstrated that SNPs associated with complex diseases can affect the expression of long non-coding RNAs (lncRNAs), suggesting that lncRNA genes are disease-susceptibility candidate genes [12].

lncRNAs are defined to be >200 nucleotides in size, contain intron/exon structure, can be expressed as alternatively spliced variants, but lack coding potential. They show, on average, expression at 2 logarithmic lower levels than protein-coding genes and it has been suggested that they can be expressed in a more cell type-specific manner than protein-coding genes [11,13,14]. Although their mechanisms of action are diverse, and not fully understood, their major function seems to be the regulation of gene expression, thus adding yet another layer of complexity to our understanding of how gene expression is regulated [15].

Recent studies have clearly demonstrated that lncRNA expression or function can be dysregulated in human diseases [12,16,17] like cancer [18-21], neurological disorders [22,23], HELLP syndrome [24], and microbial susceptibility [25]. It has also been established that lncRNAs are involved in the regulation of the immune system: in NFκB signaling, in the anti-viral response, in CD4^+^ and CD8^+^ T-cell differentiation, and in the inflammatory response [26-30]. We have recently shown that approximately 10% of AID-associated SNPs localize to lncRNA genes present in AID-associated loci [10], suggesting that the lncRNAs they encode play a role in disease etiology.

Here, we provide evidence supporting the hypothesis that lncRNA genes in AID loci may be important in disease etiology. Analyses of RNA sequencing (RNA-seq) data obtained from 11 distinct immune cell-type subsets showed enriched expression of lncRNAs located in AID loci in these cells, and allowed us to infer disease-specific immune cell subsets. To obtain more insight into the function of these lncRNAs, we performed co-expression analysis of protein-coding and lncRNA genes. This ‘guilt-by-association’ approach identified specific pathways in which AID-associated lncRNAs are involved.

## Methods

### Ethics statement

This study was approved by the Medical Ethical Board of University Medical Center Groningen (one blood sample was obtained from a healthy donor who signed an institutional review board protocol), and by the Ethics Committee of the Hospital District of Southwest Finland (naive umbilical cord blood samples from healthy neonates born in Turku University Central Hospital) in line with the guidelines of the 1975 Declaration of Helsinki. Informed consent was obtained in writing from each subject.

### Autoimmune disease locus definition

We selected all autoimmune and immune-related diseases with published Immunochip data (as of 1 June 2013) and extracted all the non-HLA signals with independent genome-wide associations (top SNPs; *P* ≤ 5 × 10^-8^). Independent association signals in regions with multiple associations were defined by applying stepwise logistic regression conditioning on the most significant variant. The Immunochip is a custom-made array containing approximately 200,000 SNPs across 186 GWAS loci for autoimmune and immune-mediated diseases. It was designed for cost-effective dense sequencing, to identify causal variants or more strongly associated variants in AID [8]. Disease-associated loci were defined as regions harboring the top SNPs and their proxy SNPs (r^2^ ≥ 0.5), which were extracted with the SNAP tool [31]. We used either the 1000 Genomes Pilot dataset [32] or the HapMap 3 (release 2) dataset [33], with the CEU population as a reference with a window of ±500 kb. For four top SNPs (rs13397, rs2097282, rs34536443, rs59466457) that were not present in both datasets, the specific disease-associated loci were defined as a 1 Mb region around the top SNP (top SNP ±500 kb; Figure S1 in Additional file 1) in analogy to what has been used in *cis*-eQTL analysis of significant associations [34]. We used the Intersect Bed method from the BEDTools suite [35] to obtain the overlapping regions between different diseases and marked them as AID shared loci.

### Collection of peripheral blood mononuclear cells and granulocytes

Venous peripheral blood (60 ml) from a healthy donor was collected in a lithium-heparin BD Vacutainer tube (BD, Franklin Lakes, NJ, USA). Peripheral blood mononuclear cells (PBMCs) were isolated by Ficoll Paque Plus (GE Healthcare Life Sciences, Uppsala, Sweden) gradient centrifugation and subjected to staining for fluorescence-activated cell sorting (FACS) analysis. The red blood cells in the pellet were lysed with monochloride solution (155 mM NH_4_Cl, 10 mM KHCO_3_, 0.1 mM Na_2_.EDTA.2H_2_O, pH 7.4) to yield the granulocyte fraction.

### Flow sorting of immune cell subsets from the PBMC fraction

The PBMCs were incubated with antibodies for 45 minutes at 4°C and sorted in six different populations on the MoFlo™ XDP flow cytometer (Beckman Coulter, Brea, CA, USA). First, lymphocytes and monocytes were separated based on forward and side scatter profiles. For further separation of lymphocytes, gates were created for CD4^-^ CD8^-^ CD56/CD16^+^ CD19^-^ (natural killer (NK) cells), CD4^-^ CD8^-^ CD56/CD16^-^ CD19^+^ (B cells), CD4^+^ CD8^-^ CD45RO^-^ (naive CD4^+^), CD4^-^ CD8^+^ CD45RO^-^ (naive CD8^+^), CD4^+^ CD8^-^ CD45RO^+^ and CD4^-^ CD8^+^ CD45RO^+^ (memory T cells) cells. Anti-CD8a-APC-eF780 and anti-CD4-eF450 were obtained from eBioscience (San Diego, CA, USA), anti-CD45RO-FITC and anti-CD19-AF700 from BD Biosciences, and anti-CD56-Pe and anti-CD16-Pe from IQ-Products (Groningen, the Netherlands).

### RNA isolation and preparation of RNA sequencing libraries

RNA was extracted from all seven immune cell types (granulocytes, monocytes, NK cells, B cells, memory T cells (both CD4^+^ and CD8^+^), naive CD4^+^ (T-helper cells) and naive CD8^+^ (cytotoxic T cells) using the MirVana RNA isolation kit (Ambion, Life Technologies, Carlsbad, CA, USA) according to the manufacturer’s instructions. We determined RNA quantity and quality using the Nanodrop 1000 Spectrophotometer (Thermo Scientific, Waltham, MA, USA) and the Experion high-sensitivity RNA analysis kit (Bio-Rad, Hercules, CA, USA), respectively. The RNA was concentrated by precipitation and re-diluted in a smaller volume. The sequencing libraries were prepared from 1 μg of total RNA using the TruSeq RNA kit (Illumina, San Diego, CA, USA) according to the manufacturer’s instructions. Each RNA library was sequenced in a single lane on the Illumina HiSeq2000 (Illumina).

### RNA sequencing of polarized human T-cell subsets derived from cord blood

Human naive umbilical cord blood CD4^**+**^ T-helper cells were isolated from healthy neonates born in Turku University Central Hospital and polarized into different T-helper cell subsets (precursor T-helper cells (ThP), primary T-helper cells (Th0) and polarized T cells (Th1, Th2)) as previously described [36]. Briefly, purified naive CD4^+^ T cells were activated with plate-bound anti-CD3 antibody (2.5 mg/ml for coating) and 500 ng/ml soluble anti-CD28 antibody (Immunotech, Marseille, France). Th1 cell polarization was initiated with 2.5 ng/ml IL-12 (R&D Systems, Minneapolis, MN, USA) and Th2 cell neutralizing antibody anti-IL-4 (1 μg/ml). To promote Th2 cell differentiation, 10 ng/ml IL-4 (R&D Systems) and Th1 cell neutralizing antibody anti-interferon gamma (1 μg/ml) was used. To obtain the Th0 population, only the neutralizing antibodies were added. At 48 hours, 40 U/ml IL-2 (R&D Systems) was added to the cultures [36]. After 7 days the polarized cells were collected and RNA was isolated using Trizol (Invitrogen, Life Technologies). The sequencing libraries were prepared from 400 ng of total RNA using the TruSeq RNA kit (Illumina) according to the manufacturer’s instructions and were sequenced on the Illumina HiSeq2000 (Illumina).

### Analysis of RNA sequencing data

The quality of the raw reads was confirmed using FastQC [37] and reads were mapped to the human reference genome (NCBI build 37) using STAR version 2.1.3 [38], allowing for two mismatches and retaining only uniquely mapping reads. The aligner was provided with a file containing junctions from Ensembl GRCh37.65. Reads that corresponded to flag 1796 in the bam alignment file (flag 1796: read unmapped, not primary alignment, read fail quality check, read is PCR or optical duplicate) were filtered out. To estimate expression levels in RNA deep sequencing data, the number of reads that overlapped with exons from known transcripts (as described in Gencode version 14 [14]) by no less than 30% of the read’s length were quantified using the IntersectBed tool from the BEDTools suite [35]. Subsequently, the reads were normalized, and normalized expression RPKM (reads per kilobase per million mapped reads) values were calculated using the formula RPKM_g_ = 10^9^ × (C_g_/(N × L_g_)) [39], where C_g_ is the number of reads that map into the exons of gene g; L_g_ is the length of the exons of gene g; and N is the total number of mapped reads for this sample. RPKM values for all Gencode version 14 genes were computed at the gene levels obtained for all 11 immune cell types, respectively. Gencode version 14 data [14] were used to annotate these regions with protein-coding and lncRNA genes using the IntersectBed tool from BEDTools suite [35]. Circular diagrams showing the genes shared between the various autoimmune diseases were produced using Circos [40].

Differences in expression between AID- or disease-specific loci and the whole Gencode reference were tested using the two-tailed Fisher’s exact test, and the *P*-values were corrected for multiple testing with the Bonferroni correction. The statistically significant thresholds for differentially expressed genes in seven peripheral immune cell types were *P* ≤ 0.007 (level of significance (α) = 0.05), *P* ≤ 0.001 (α = 0.01), and *P* ≤ 0.0007 (α = 0.005), and in four cord blood CD4^+^ T-cell lineages they were *P* ≤ 0.012, *P* ≤ 0.002, and *P* ≤ 0.0012, respectively.

The normalized gene expression values (RPKM) were log10 transformed. For zero expression (0 RPKM) a 0.000001 value was added to the RPKM value and log10 transformed. Heat maps of the transformed RPKM data were created in Gene-E and unsupervised hierarchical clustering of the samples was performed using the ‘average linkage’ clustering method with the Euclidean distance metric [41].

The RNA sequencing data from this study are available from Gene Expression Omnibus [42], accession number GSE62408.

## Results

### Selection of AID phenotypes

In order to investigate the shared genetics of autoimmune and immune-related diseases, we selected eight different AID for which dense-mapped Immunochip data were available (per 1 June 2013): autoimmune thyroid disease [43], celiac disease (CeD) [44], inflammatory bowel disease (IBD) [45], juvenile idiopathic arthritis (JIA) [46], primary biliary cirrhosis (PBC) [47], psoriasis (PS) [48], primary sclerosing cholangitis (PsCh) [49] and rheumatoid arthritis (RA) [50]. We sub-divided IBD loci into Crohn’s disease (CD)-specific loci, ulcerative colitis (UC)-specific loci, and CD-UC shared loci (IBD shared) to reveal phenotype-specific features. Autoimmune thyroid disease was excluded from further analysis since only two SNPs reported in this study [43] passed the stringent genome-wide *P*-value cutoff (*P* ≤ 5 × 10^-8^). We thus had nine disease phenotypes to analyze: CD, CeD, IBD shared, JIA, PBC, PS, PsCh, RA, and UC.

### Locus definition and overlap between other AIDs

After selecting the disease phenotypes, we defined the loci associated with the individual phenotypes (Additional file 1), resulting in a total number of 284 loci (Table 1; Additional file 2). Of these 284 loci, 119 loci overlapped partly or completely in two or more AID and are referred to as ‘AID shared loci’ (Additional file 3). Next, we examined whether the size of the shared loci was related to the number of diseases it was associated with, but we observed no enrichment of the number of AIDs in any specific size class (Figure S2A,B in Additional file 4).

**Table 1:** **Overview of the nine autoimmune diseases (AIDs) included in this study**

**Autoimmune disease**	**Number of Immunochip top SNPs**	**1000 genomes pilot project**			**HapMap 3**		**SNP ±500 kb**		**Number of genes (Gencode version 14)**		
		**SNPs found**	**Proxy SNPs (r** ^**2**^ **= 1)**	**Average locus size (bp)**	**SNPs found**	**Average locus size (bp)**	**SNPs found**	**Locus size (bp)**	**Protein-coding**	**lncRNA**	**Reference**
**CD**	29	29	338	123,953	-	-	-	-	50	14	[45]
**CeD**	32	30	246	125,799	-	-	2	1,000,000	127	37	[44]
**IBD (shared)**	97	95	984	139,174	2	172,626	2	1,000,000	254	107	[45]
**JIA**	16	15	112	152,964	-	-	1	1,000,000	73	20	[46]
**PBC**	18	16	143	176,717	1	859,903	2	1,000,000	90	38	[47]
**PS**	33^a^	32	287	111,617	-	-	1	1,000,000	131	48	[48]
**PsCh**	12	12	81	227,273	-	-	-	-	50	17	[49]
**RA**	26	23	132	96,343	-	-	3	1,000,000	127	36	[50]
**UC**	22	22	198	127,674	-	-	-	-	48	24	[45]
**Total**	**285**	**274**	**2,521**	**142,390**	**3**	**516,265**	**11**	**1,000,000**	**950**	**341**	

CD, Crohn’s disease-specific; CeD, celiac disease; IBD (shared), inflammatory bowel disease shared by Crohn’s disease and ulcerative colitis; JIA, juvenile idiopathic arthritis; PBC, primary biliary cirrhosis; PS, psoriasis; PsCh, primary sclerosing cholangitis; RA, rheumatoid arthritis; UC, ulcerative colitis-specific. Each disease is characterized by the number of genome-wide significant SNPs associated with the disease phenotype, the number of disease loci, and the genes located inside them. We subdivided inflammatory bowel disease loci into Crohn’s disease-specific loci (CD), ulcerative colitis-specific loci (UC), and CD-UC shared loci (IBD shared) to reveal phenotype-specific features. ^a^Psoriasis (PS) was associated with 33 genome-wide significant SNPs. After defining the PS disease loci, we discovered that one locus (chr.19: 10,745,764-10,894,728) is located within a bigger 1 Mb locus (chr.19: 9,963,118-10,963,118), resulting in 32 PS loci and making the total number of AID loci 284. In summary, the total number of AID-associated SNPs was 285 and these led to 284 AID loci. After all the disease loci annotation with genes from the reference dataset (Gencode version 14), we discovered 950 protein-coding genes in total but 186 of them were shared between at least two disease phenotypes (AID-shared coding genes) and 626 were unique across all diseases (AID-coding genes). For lncRNAs, the total number of genes was 341, with 61 shared (AID-shared lncRNAs) and 240 unique genes (AID-lncRNAs).

### Annotation of protein-coding and non-coding genes in AID loci

To identify lncRNAs and protein-coding genes localized in selected loci, we annotated all 284 AID loci with Gencode V14 data. This resulted in 240 lncRNAs and 626 protein-coding genes in these loci as shown in Table 1. More detailed information about the specific genes transcribed in each AID locus is provided by disease phenotype (Additional file 5) and by chromosome coordinates (Additional file 6). We observed a lncRNA to protein-coding gene ratio of approximately 1:3 in all but one disease (UC-specific loci were represented by a 1:2 ratio), which is nearly double the 1:1.6 genome-wide ratio calculated from using all 12,933 lncRNAs and 20,074 protein-coding genes (Table 1).

**Figure 1:**
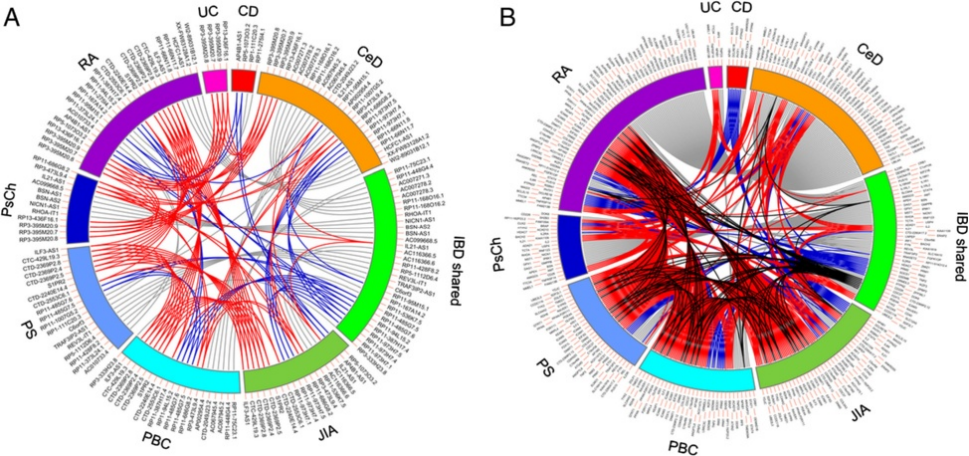
**Circular diagrams showing the genes shared between nine autoimmune diseases.** The nine diseases are shown on the outer circle in colored bands, with their abbreviated names. The ribbons depicting the shared genes are colored according to the number of disease phenotypes they are shared by (grey, two AIDs; blue, three AIDs; red, four AIDs; black, five AIDs). **(A)** lncRNA genes and **(B)** protein-coding genes shown in this figure include genes specific to Crohn’s disease (CD), celiac disease (CeD), inflammatory bowel disease, shared by Crohn’s disease and ulcerative colitis (IBD shared), juvenile idiopathic arthritis (JIA), primary biliary cirrhosis (PBC), psoriasis (PS), primary sclerosing cholangitis (PsCh), rheumatoid arthritis (RA), and ulcerative colitis specific (UC) genes. These two plots are presented at higher resolution in Additional files 8 and 9).

Since we observed frequent overlap at the disease locus level, we then investigated the inter-disease overlap at the gene level as well (Figure 1). As expected, the profile for the number of shared protein-coding genes was almost identical to that found for the shared lncRNAs, suggesting that lncRNAs might be similar in their level of importance to that of protein-coding genes in AIDs (Additional files 7, 8, 9, 10, 11, 12, and 13). For example, the highest number of shared lncRNAs (11), as well as the highest number of protein-coding genes (51), was observed between RA and CeD (representing 31% of all RA lncRNAs and 30% of all CeD lncRNAs versus 40% of all RA protein-coding genes and 40% of all CeD protein-coding genes) (Additional files 7, 8, and 9), which agrees with previous findings from the literature [51].

### Expression pattern of lncRNA and protein-coding genes in distinct immune cell subsets

Immune cells are the major ‘disease effector’ cell types in AIDs and previous studies have reported a critical role for T-cell differentiation and enrichment of causal genes for Th1 and Th2 pathways [52-55]. Since data on lncRNA genes are lacking, we investigated the expression levels of AID locus-encoded genes in seven circulating immune cell subsets and in four cell types during CD4^+^ T-cell differentiation using the RNA-sequencing data.

On average, the total number of sequencing reads per sample was 137,411,294 for the seven immune cell subsets and 199,151,275 reads for the polarized human T-cell subsets generated from cord blood. Approximately 88% of the reads were mapped to the reference genome on average.

**Figure 2:**
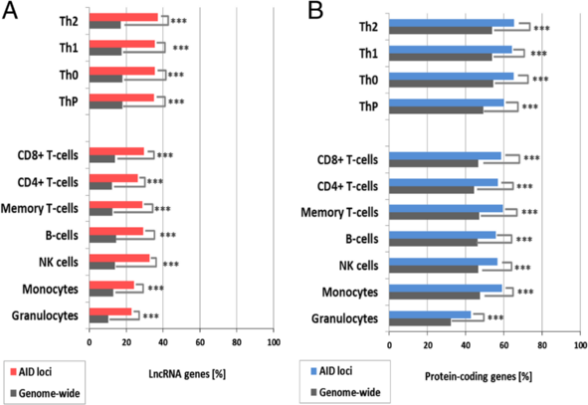
**Proportion of genes expressed in different immune cells. (A)** The number of lncRNA genes expressed (>2 RPKM) as a percentage of all lncRNA genes genome-wide (n = 12,933) or as a percentage of all lncRNAs located in autoimmune disease loci (n = 240 genes). **(B)** The data for the protein-coding genes genome-wide (n = 20,074) and the ones in AID loci (n = 626). Statistically significant enrichments (*P*-values) after Bonferroni correction for multiple testing are denoted by asterisks to show the different levels of significance (*α < 0.05; **α < 0.01; ***α < 0.005).

Analyzing the expression data genome-wide, we see for lncRNAs that, on average, 15% of all genes (1,881 out of 12,933) are expressed in the 11 cell types we investigated (Figure 2A). If we focus only on the expressed lncRNAs from the AID loci and compare them to the expressed lncRNAs from the whole genome (15%), we see a two-fold increase to 32%, on average, representing 73 out of all 240 AID lncRNA genes. As can be seen from Figure 2A, the range of gene expression in seven circulating immune cell types is lower (23 to 33%) compared with four types of differentiated CD4^+^ T cells (35 to 37%). Consistent with this observation, in both datasets, we see similar enrichments of expression of protein-coding genes encompassed within the AID loci (61%, 380 genes) compared with all Gencode protein-coding genes (47%, 9,526 genes) (Figure 2B). All the reported differences in expression are statistically significant (α < 0.005) after Bonferroni correction for multiple testing as shown in Figure 2 and Additional file 14.

To determine which immune cell types are involved in a specific disease, we then investigated associations between lncRNA expression profiles and disease-specific loci for each individual disease (Additional files 15 and 16). Firstly, for four diseases, we observed enrichment of differentially expressed lncRNAs between those in the disease loci and all Gencode lncRNAs (α < 0.005) in three circulating immune cell types (NK cells for IBD, JIA, PBC, PS; memory and CD8^**+**^ T cells for JIA, PBC, PS, RA; Figure S6A in Additional file 11). Secondly, for five diseases (IBD shared, JIA, PBC, PS (α < 0.01); RA (α < 0.05)) enrichment was observed for all four CD4^+^ T-cell subsets tested (Figure S6B in Additional file 11). Thirdly, the lncRNAs in the PS loci were differentially expressed in all 11 cell types (α < 0.005) (Figure S7F in Additional file 12; Figure S8F in Additional file 13), suggesting that these abundant lncRNAs in the PS loci may act in a less cell type-specific manner but a more disease-specific one. As shown in Additional file 11, we observed an interesting but expected pattern of enrichment, in which protein-coding genes in AID loci were significantly more expressed in all the tested cell types than the protein-coding genes from the whole Gencode dataset (Figure S6C,D in Additional file 11). Similar enrichment was also seen for lncRNAs, although the enrichment was more cell type-specific (Figure S6A in Additional file 11), supporting the characteristic attribute of lncRNAs as cell type-specific transcripts.

### Gene expression distribution and levels in immune cell subsets

**Figure 3:**
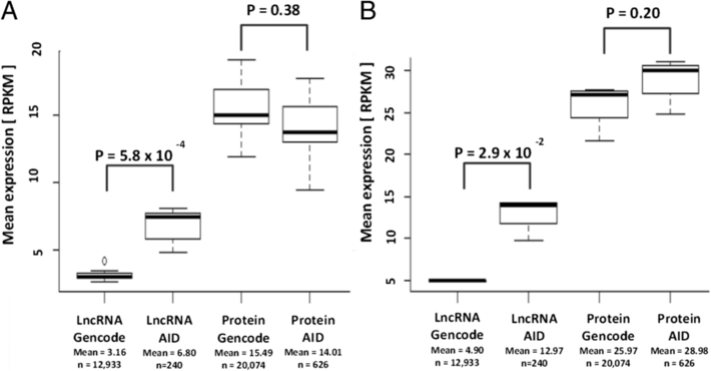
**Mean expression distribution of lncRNAs and protein-coding genes. (A)** Comparison of lncRNA expression genome-wide (LncRNA Gencode, n = 12,933) with expression of lncRNAs located in AID loci (LncRNA AID, n = 240) and the expression of protein-coding genes genome-wide (Protein Gencode, n = 20,074) with the expression of protein-coding genes located in AID loci (Protein AID, n = 626) in seven populations of peripheral blood leukocytes. **(B)** Similar data for the T-helper cell populations derived from cord blood. Differences in the means of expression levels between the two groups (disease loci (AIDs) versus genome-wide (Gencode)) were tested for significance using the Wilcoxon rank-sum test.

To gain a detailed picture of lncRNA and protein-coding gene expression profiles in our data, we computed the gene expression distribution separately for both datasets (Figure 3). Our data confirm that all Gencode lncRNA are, in general, significantly less expressed than all protein-coding genes (approximately five-fold lower in both circulating (*P* = 0.00058) or T-helper cell subsets (*P* = 0.029) (Figures 3A,B). Next, we focused our attention on the gene expression distribution in AID loci and the differences compared with the whole genome. We computed the expression distribution of genes in AID loci and compared it with the expression distribution of all Gencode lncRNA and coding genes. Figure 3 shows that lncRNAs associated with AID loci display an approximately 2.5-fold higher mean expression distribution than all lncRNAs. In contrast, the protein-coding genes in the AID loci displayed similar expression distributions compared with all the coding genes in the Gencode dataset (Figure 3).

Comparing the mean expression levels of lncRNAs versus protein-coding genes in AID loci revealed only an approximately two-fold lower expression of AID lncRNAs (lncRNAs: in circulating peripheral cells = 6.80 RPKM; in cord blood T-helper cells = 12.97 RPKM; coding genes: in circulating cells = 14.01 RPKM; T-helper cells = 28.98 RPKM). This suggests that lncRNAs in disease-associated loci are expressed to higher levels than previously assumed and that they do so in cell types functionally involved in the disease (Figure 3). Together, these findings suggest an important, cell type-specific role for lncRNA genes located in AID loci in immune cell biology and AIDs.

### Analysis of lncRNA expression profiles

To examine the cell type-specific expression patterns of individual lncRNAs, we created heat maps of all 240 AID lncRNAs (Additional file 6) in the 11 cell types investigated (Figure S7A in Additional file 15) and observed small cell type-specific clusters of lncRNAs. For instance, seven lncRNAs (*RP11-324I22.2* (IBD), *RP5-1011O1.2* (CeD), *AC074391.1* (IBD), *AC012370.2* (IBD), *ALG9-IT1* (PsCh), *BSN-AS1* (IBD, PsCh), *CTC-349C3.1* (UC)) were only expressed in four T-helper cell subtypes (ThP, Th0, Th1 and Th2), whereas one lncRNA (*CTD-2113 L7.1* (PBC)) was expressed in all the T cells investigated. Two lncRNAs (*AP002954.3* (CeD) and *RP11-84D1.2* (PS)) were detected in CD4^+^ T cells, CD8^+^ T cells, ThP, Th0, Th1 and Th2 cells, but not in memory T cells.

## Discussion

Interpreting the mechanisms of action of disease-associated SNPs identified by GWAS is a challenge because the vast majority of them are located in non-coding regions that might play a more regulatory role. An extra complication is the recent discovery of a new class of regulatory RNAs, the lncRNAs. It has now been recognized that many regions previously designated as ‘gene deserts’ actually harbor lncRNA genes. In this study, we set out to investigate the nature of lncRNAs present in AID loci in more detail, by analyzing gene expression across 11 distinct immune cell types. We assumed that lncRNAs that are highly expressed in particular cell types are functionally active [11] and that they can be used to prioritize disease-specific cell types. We observed an expression enrichment of AID locus genes (both protein-coding and lncRNAs) and confirmed the cell type-specific pattern of lncRNAs for AID loci. For example, there are almost no publications on the involvement of specific immune cells in UC versus CD, while our data suggest that NK cells and granulocytes are involved in both UC and CD (that is, in IBD-shared loci), whereas T and B cells are associated specifically with UC. In the case of RA, AID lncRNAs were more abundant in the T-cell compartment (memory T, naive CD8^+^ T, ThP, Th0, Th2 cells), which agrees with a study based on a statistical approach to murine immune cells demonstrating enrichment of protein-coding genes in CD4^+^ memory T cells [56]. We observed no expression enrichment of CeD genes in any of the cell types tested, suggesting that the main effector cell type involved in the pathophysiology of CeD might not have been represented by the cell types present in our panel of cells. Gluten-reactive CD4^+^ T-cell clones or the autoreactive CD8^+^ T cells (intraepithelial cytotoxic T lymphocytes) that have infiltrated into the epithelium in the small intestine of CeD patients are thought to be the key effector cells and these cells should be included in future studies [5].

**Figure 4:**
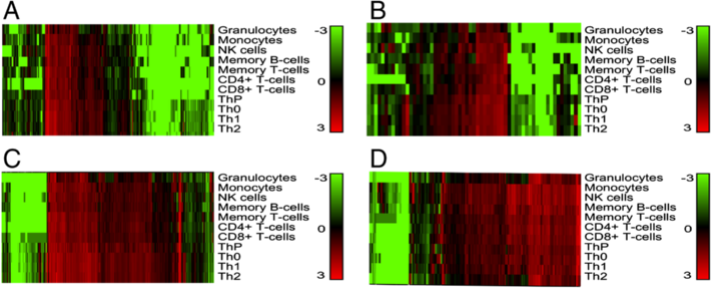
**RNA sequencing analysis of gene expression in seven peripheral blood leukocyte populations and four T-helper cell populations from cord blood.** The heat maps show the expression of all genes located in AID loci: **(A)** lncRNAs; **(B)** protein-coding genes and AID genes shared by at least two diseases: **(C)** lncRNAs; **(D)** protein-coding genes. Unsupervised hierarchical clustering analysis of gene expression profiles of all 11 cell types (granulocytes, monocytes, NK cells, B cells, memory T cells (both CD4^+^ and CD8^+^), naive CD4^+^and naive CD8^+^ T cells (cytotoxic T cells), precursor T-helper cells (ThP), primary T-helper cells (Th0), and polarized T cells (Th1, Th2)). Heat maps represent log10 intensity values. In the color scheme, saturated red indicates three-fold up-regulation, saturated green indicates three-fold down-regulation, and black indicates unchanged expression.

**Figure 5:**
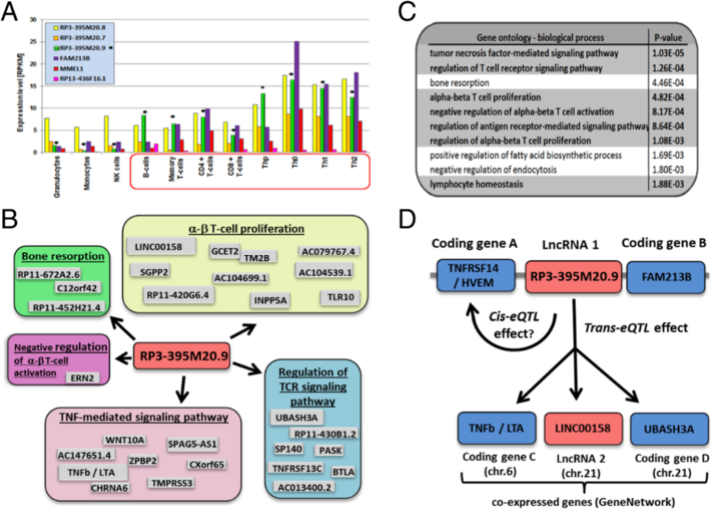
**An example of analyzing an autoimmune disease locus by pathway analysis approaches. (A)** Expression levels of protein-coding transcripts (FAM213B, MMEL1) and lncRNA genes (RP3-395 M20.8, RP3-395 M20.7, RP3-395 M20.9, RP13-436 F16.1) located in the MMEL1 locus associated with four AIDs. The arrows pinpoint the data for *RP3-395 M20.9*. **(B)** Genes co-expressed with *RP3-395 M20.9* are grouped in five differently colored segments corresponding to the pathways predicted by GeneNetwork. **(C)** The top 10 Gene Ontology (GO) biological processes predicted to be associated with the genes co-expressed with RP3-395 M20.9 are shown. **(D)** This schema shows a hypothetical mechanism of action of *RP3-395 M20.9.* The disease-associated SNP is located between protein-coding gene A (tumor necrosis factor receptor superfamily, member 14 (TNFRSF14, HVEM)) and lncRNA 1 (RP3-395 M20.9). This SNP only affects *RP3-395 M20.9* directly. Two protein-coding genes (tumor necrosis factor beta/lymphotoxin alpha (TNFb/LTA) on chromosome 6, and UBASH3A on chromosome 21) and one lncRNA (LINC00158 on chromosome 21) are co-expressed with *RP3-395 M20.9*, which could be due to *trans*-regulation of these genes by *RP3-395 M20.9*. A hypothetical *cis*-effect of lncRNA 1 (*RP3-395 M20.9*) on protein-coding gene TNFRSF14/HVEM in the same locus on chromosome 1 is also mentioned.

Many of the protein-coding genes in the AID loci are known to play important roles in immune cell development and/or function, but relatively little is known about the role of lncRNAs in the immune system [25,28-30]. Co-expression analysis of transcripts is a promising strategy to predict the function of lncRNA genes using a ‘guilt-by-association’ approach. To date, most co-expression data have been provided by gene expression microarrays that contain only a small subset of probes to lncRNAs [12]. Despite this limitation, we used GeneNetwork [57], which uses co-expression data to predict pathways and tissues in which the query lncRNA could be involved. From our 240 AID lncRNAs (Additional file 6; Figure S4A in Additional file 8; a higher resolution figure is provided in Figure S9A in Additional file 17), we selected those that were associated with at least two AIDs (Figure 4C; Additional file 12; Figure S9C in Additional file 17). Of these 61 AID-lncRNAs, 9 were present in GeneNetwork, which we then used to obtain Gene Ontology (GO) terms associated with specific co-expression profiles (Additional file 18) [58]. Based on these results, we could show, for instance, that lncRNA *RP3-395 M20.9* is co-expressed with genes known to be involved in T- and B-cell biology (Figure 5B). It is located in a locus shared by CeD, PsCh, RA, and UC, and is abundant in monocytes and B and T lymphocytes (B cells, memory T cells, CD4^+^ T cells, and in all four cord blood T-helper cells) (Figure 5A). Seven of the top 10 GO biological processes predicted to be associated with genes co-expressed with this lncRNA contained ‘tumor necrosis factor (TNF) pathway’ or ‘T-cell/lymphocyte event’ in their description (Figure 5C; Additional file 18), confirming our results from expression analysis. Figure 5D visualizes the connection between the lncRNA *RP3-395 M20.9* and the co-expressed protein-coding and non-coding genes proposed by GeneNetwork. Now that the pathways and disease-relevant cell types in which this lncRNA is involved are known, it is easier to design appropriate functional follow-up studies.

Here we show, for the first time, that AID lncRNA expression profiles predict cell type specificity better than AID protein-coding genes. Our findings have implications for identifying relevant disease-specific cell types, not only for AIDs but also for other complex disorders. We realize that by defining the disease loci, we may have excluded a few causal genes, since they can be located outside these loci due to more complex gene regulation. To address this possibility, the next logical step would be to perform eQTL analysis across a wide region and to analyze both protein-coding and lncRNA genes. Preliminary results from such an eQTL analysis of RNA sequencing data generated from 673 whole blood samples suggest that the majority of AID lncRNA eQTLs are *cis-*eQTLs (I Ricaño-Ponce *et al.*, personal communication). Ideally, the proposed eQTL analyses should be performed using RNA sequencing data obtained from individual immune cell subsets rather than from whole blood, as is currently often the case. As such datasets are likely to become available in the near future, they will allow better co-expression-based pathway analyses and, subsequently, a more precise prediction of lncRNA function.

In order to test our hypothesis of the involvement of lncRNAs in immune cell signaling, laboratory-based experiments need to be performed to validate the *in silico* predictions and to elucidate the mechanism by which the lncRNAs regulate the expression of protein-coding genes. We were able to find lncRNA-protein-coding gene pairs present in a single AID locus and these pairs are co-regulated in specific immune cell types. For example, the IL21-IL21-AS1 locus, associated with CeD, JIA, PsCh, and IBD, contains four protein-coding genes (KIAA1109, *ADAD1*, *IL2*, *IL21*) and one lncRNA (IL21-AS1). IL21-AS1 exhibits a clear co-expression profile with IL-21 in Th1 cells, where the level of IL21-AS1 is similar to IL-21 (Additional file 19). We realize that enrichment statistics or gene co-expression are not conclusive with regard to causality and that functional studies knocking-down protein-coding and/or lncRNA genes, followed by rescuing experiments, are necessary.

## Conclusions

Our results suggest that immune cell-specific expression or function of lncRNAs is important in the etiology of auto-immune diseases, possibly by regulating the expression of proteins critical for proper immune function.

## Additional files

Additional file 1: Figure S1. A schematic illustration of our procedure to define autoimmune disease loci. The independent association signals in regions with multiple associations were defined using stepwise logistic regression conditioning on the most significant variant. Disease-associated loci were defined as regions containing the top SNP and its proxy SNPs (r^2^ ≥ 0.5) selected either from the 1000 Genomes Pilot (1000G) dataset or from the HapMap 3 dataset [32,33]. The disease locus was defined as a region with a fixed size of 1 Mb (top SNP ±500 kb) when the top SNP was absent from 1000G and HapMap3. We defined a disease locus as having a fixed 1 Mb size only in those cases that the disease-associated SNP was absent from the 1000 Genomes pilot and the HapMap 3 datasets. This was the case for only four AID loci: one shared by five AIDs (JIA, PBC, PS, RA, IBD-shared), one shared by RA and IBD; one shared by CeD and RA; and one CeD-only locus. Additional file 2: Table S1. List of loci associated with nine autoimmune diseases. For each disease phenotype, the table shows a list of loci including additional information like chromosome position (Chr), genome coordinates of the beginning in human genome build 19 (Start position (hg19)) and of the end (End position (hg19)) of each locus, the size of the locus in base pairs (Locus size [bp]) and the label of the disease loci (AID loci ID). Additional file 3: Table S2 Visualization of AID loci shared between autoimmune diseases. In this table we defined AID shared loci as those with two or more diseases overlapping with the locus. Each AID shared locus includes information about the number of AIDs overlapping this region, the locus size in base pairs (Size [bp]) and the list of AID loci IDs. Data are sorted based on the highest number of AIDs in one locus. Additional file 4: Figure S2. Absence of a relationship between locus size and the number of autoimmune diseases (AIDs) associated with those loci. **(A)** Number of diseases sharing one AID locus (x-axis) versus locus size distribution (y-axis). All the AID loci were grouped in five differently colored segments based on the size range of each locus (<10 kb; 10 to 250 kb; 250 to 500 kb; 500 kb to 1 Mb; 1 Mb). **(B)** The characteristics and distribution of AID loci. The number of AIDs associated with a given locus is plotted on the x-axis. The green bars represent the average locus size in base pairs (kb) on the left-hand y-axis. The red line corresponds to the number of loci in each group of loci shared by a certain number of AIDs on the right-hand y-axis. Additional file 5: Table S3. AID loci annotated with genes and listed by disease phenotype. Each AID loci is annotated by protein-coding and lncRNA genes either from Gencode version 14 reference (GencodeV14-protein_coding (violet), GencodeV14 all_LncRNAs (green)) or from the Human long intergenic non-coding RNAs (lincRNAs) catalog (Cabili_LincRNAs (orange)). Each gene is characterized by its chromosome number (Chr), the genome coordinates of the beginning in human genome build 19 (START (hg19)) and end (END (hg19)), the gene name (Gene ID (ENCODE/Cabili)), the identification number used in the Ensembl database (Gene ID (Ensembl)), the strand specification (Strand) and color coded information about the gene type (Gene type). Additional file 6: Table S4. AID loci annotated with genes and listed by chromosome coordinates. Each gene from previously annotated AID loci using Gencode V14 (GencodeV14-protein-coding (violet), GencodeV14 all_LncRNAs (green)) and lincRNAs catalog (Cabili_LincRNAs (orange)) is characterized by its chromosome number (Chr), the genome coordinates of the beginning in human genome build 19 (START (hg19)) and end (END (hg19)), the gene name (Gene ID (ENCODE/Cabili)), identification number used in the Ensembl database (Gene ID (Ensembl)), the strand specification (Strand), the color coded information about the gene type (Gene type) and the listed names of AID loci in which a particular gene is located. Genes highlighted in dark green are shared between two or more AIDs. Additional file 7: Figure S3. Heat map visualization of overlapping genes and loci between the autoimmune diseases studied in this paper. **(A)** lncRNA genes; **(B)** protein-coding genes; **(C)** loci shared between the pairs of diseases. CD, Crohn’s disease-specific; CeD, celiac disease; IBD-shared, inflammatory bowel disease shared by Crohn’s disease and ulcerative colitis; JIA, juvenile idiopathic arthritis; PBC, primary biliary cirrhosis; PS, psoriasis; PsCh, primary sclerosing cholangitis; RA, rheumatoid arthritis; UC, ulcerative colitis-specific. In the color scheme, red indicates the highest number of shared properties, and white indicates the lowest number of shared properties (see legend under each heat map). Additional file 8: Figure S4. Circular diagram showing the disease-specific lncRNA genes shared between nine autoimmune diseases in detail. The nine diseases are shown on the outer circle in colored bands, with their abbreviated names. The ribbons depicting the shared genes are colored according to the number of disease phenotypes they are shared by (grey, two AIDs; blue, three AIDs; red, four AIDs). The lncRNA genes shown in this figure include genes specific to Crohn’s disease (CD), celiac disease (CeD), inflammatory bowel disease, shared by Crohn’s disease and ulcerative colitis (IBD_shared), juvenile idiopathic arthritis (JIA), primary biliary cirrhosis (PBC), psoriasis (PS), primary sclerosing cholangitis (PsCh), rheumatoid arthritis (RA), ulcerative colitis specific (UC) genes. Additional file 9: Figure S5. Circular diagram showing the disease-specific protein-coding genes shared between nine autoimmune diseases in detail. The nine diseases are shown on the outer circle in colored bands, with their abbreviated names. The ribbons depicting the shared genes are colored according to the number of disease phenotypes they are shared by (grey, two AIDs; blue, three AIDs; red, four AIDs; black, five AIDs). The protein-coding genes shown in this figure include genes specific to Crohn’s disease (CD), celiac disease (CeD), inflammatory bowel disease, shared by Crohn’s disease and ulcerative colitis (IBD_shared), juvenile idiopathic arthritis (JIA), primary biliary cirrhosis (PBC), psoriasis (PS), primary sclerosing cholangitis (PsCh), rheumatoid arthritis (RA), ulcerative colitis specific (UC) genes. Additional file 10: Table S5. List of protein-coding genes shared between diseases. For each disease a list of shared protein-coding genes is provided, as well as information of which disease the gene is shared with. CD, Crohn’s disease-specific; CeD, celiac disease; IBD-shared, inflammatory bowel disease shared by Crohn’s disease and ulcerative colitis; JIA, juvenile idiopathic arthritis; PBC, primary biliary cirrhosis; PS, psoriasis; PsCh, primary sclerosing cholangitis; RA, rheumatoid arthritis; UC, ulcerative colitis-specific. Additional file 11: Table S6. List of lncRNA genes shared between diseases. For each disease a list of shared lncRNAs (including information about the Gencode sub-classification) is provided, as well as information of which disease the gene is shared with. CD, Crohn’s disease-specific; CeD, celiac disease; IBD-shared, inflammatory bowel disease shared by Crohn’s disease and ulcerative colitis; JIA, juvenile idiopathic arthritis; PBC, primary biliary cirrhosis; PS, psoriasis; PsCh, primary sclerosing cholangitis; RA, rheumatoid arthritis; UC, ulcerative colitis-specific. Additional file 12: Table S7. List of lncRNA genes shared by two or more AIDs. All AID-shared lncRNAs (LncRNA ID_GencodeV14, n = 61) are characterized by an identification number used in the Ensembl database (Gene_ID_Ensembl), chromosome number (Chr), genome coordinates of the beginning in human genome build 19 (Start (hg19)) and end (End (hg19)), the strand specification (Strand) and the list of names of AIDs (CD, Crohn’s disease-specific; CeD, celiac disease; IBD-shared, inflammatory bowel disease shared by Crohn’s disease and ulcerative colitis; JIA, juvenile idiopathic arthritis; PBC, primary biliary cirrhosis; PS, psoriasis; PsCh, primary sclerosing cholangitis; RA, rheumatoid arthritis; UC, ulcerative colitis-specific) in which a particular gene is located. Additional file 13: Table S8. List of protein-coding genes shared by two or more AIDs. All AID shared protein-coding genes (Prot-coding ID_GencodeV14, n = 186) are characterized by identification number used in the Ensembl database (Gene ID (Ensembl)), the chromosome number (Chr), the genome coordinates of the beginning in human genome build 19 (START (hg19)) and end (END (hg19)), the strand specification (Strand) and the list of names of AIDs (CD, Crohn’s disease-specific; CeD, celiac disease; IBD-shared, inflammatory bowel disease shared by Crohn’s disease and ulcerative colitis; JIA, juvenile idiopathic arthritis; PBC, primary biliary cirrhosis; PS, psoriasis; PsCh, primary sclerosing cholangitis; RA, rheumatoid arthritis; UC, ulcerative colitis-specific) in which a particular gene is located. Additional file 14: Figure S6. Cell type-specific expression enrichment of genes located in disease loci. For each disease, we compared the proportion of expressed genes (>2 RPKM) in the whole genome (Gencode version 14) with the proportion of expressed genes located within disease-specific loci, similar to what we had done for all the AID loci together (Figure 2), and tested for differences using two-tailed Fisher’s exact test. Statistically significant enrichment (*P*-values after Bonferroni correction for multiple testing) is denoted by color and plus symbols to show the different levels of significance as defined in the legend. (A-D) Expression enrichment for lncRNAs (top) and protein-coding genes (bottom) in seven peripheral leukocyte (A,C) and four cord blood T-helper (B,D) cell types were calculated for all AID loci together (all AID) as well as separately for each disease (CD, Crohn’s disease-specific; CeD, celiac disease; IBD-shared, inflammatory bowel disease shared by Crohn’s disease and ulcerative colitis; JIA, juvenile idiopathic arthritis; PBC, primary biliary cirrhosis; PS, psoriasis; PsCh, primary sclerosing cholangitis; RA, rheumatoid arthritis; UC, ulcerative colitis-specific). Additional file 15: Figure S7. Proportion of lncRNA genes expressed in seven peripheral blood leukocyte populations and four cord blood T-helper cell populations, per disease. For each disease, we compared the proportion of expressed lncRNAs (>2 RPKM) in the whole genome (genome-wide, gray) with the proportion of expressed disease-specific lncRNAs (red), and tested for differences using two-tailed Fisher’s exact test. Statistically significant enrichments (*P*-values) after Bonferroni correction for multiple testing are denoted by asterisks to show the different levels of significance (*α < 0.05; **α < 0.01; ***α < 0.005). **(A)** Crohn’s disease-specific; **(B)** celiac disease; **(C)** inflammatory bowel disease shared by Crohn’s disease and ulcerative colitis; **(D)** juvenile idiopathic arthritis; **(E)** primary biliary cirrhosis; **(F)** psoriasis; **(G)** primary sclerosing cholangitis; **(H)** rheumatoid arthritis; **(I)** ulcerative colitis-specific. Additional file 16: Figure S8. Proportion of protein-coding genes expressed in seven peripheral blood leukocyte and four cord blood T-helper cell populations, per disease. For each disease, we compared the proportion of expressed coding genes (>2 RPKM) in the whole genome (genome-wide, gray) with the proportion of expressed disease-specific coding genes (red), and tested for differences using two-tailed Fisher’s exact test. Statistically significant enrichments (*P*-values) after Bonferroni correction for multiple testing are denoted by asterisks to show the different levels of significance (*α < 0.05; **α < 0.01; ***α < 0.005). **(A)** Crohn’s disease-specific; **(B)** celiac disease; **(C)** inflammatory bowel disease shared by Crohn’s disease and ulcerative colitis; **(D)** juvenile idiopathic arthritis; **(E)** primary biliary cirrhosis; **(F)** psoriasis; **(G)** primary sclerosing cholangitis; **(H)** rheumatoid arthritis; **(I)** ulcerative colitis-specific. Additional file 17: Figure S9. RNA sequencing analysis of gene expression in seven peripheral blood leukocyte and four cord blood T-helper cell populations. Each panel is shown as a separate file with a higher resolution so that the gene IDs can be read easily. (A-D) The heat maps show expression of all genes located in AID loci ((A) lncRNAs, (B) protein-coding genes) and AID genes shared by at least two diseases ((C) lncRNAs, (D) protein-coding genes) in all 11 cell types (granulocytes, monocytes, NK cells, B cells, memory T cells (both CD4^+^ and CD8^+^), naive CD4^+^and naive CD8^+^ T cells (cytotoxic T cells), precursor T-helper cells (ThP), primary T-helper cells (Th0) and polarized T cells (Th1, Th2)). In the color scheme, saturated red indicates three-fold up-regulation, saturated green indicates three-fold down-regulation, and black indicates unchanged expression. **(A)** RNA sequencing analysis of gene expression in seven peripheral blood leukocyte and four cord blood T-helper cell populations - 240 AID lncRNAs. **(B)** RNA sequencing analysis of gene expression in seven peripheral blood leukocyte and four cord blood T-helper cell populations - 626 AID protein-coding genes. **(C)** RNA sequencing analysis of gene expression in seven peripheral blood leukocyte and four cord blood T-helper cell populations - 61 lncRNAs shared between at least two AIDs. **(D)** RNA sequencing analysis of gene expression in seven peripheral blood leukocyte and four cord blood T-helper cell populations - 186 protein-coding genes shared between at least two AIDs. Additional file 18: Table S9. Results from pathway analysis using the GeneNetwork tool of nine AID shared lncRNAs. Nine out of 61 AID shared lncRNAs were present in the GeneNetwork database. Each lncRNA gene (LncRNA ID_GencodeV14) is characterized by an identification number used in the Ensembl database (Gene_ID_Ensembl), its chromosome number (Chr), the genome coordinates of the beginning in human genome build 19 (START (hg19)) and end (END (hg19)), the strand specification (Strand) and the list of names of AIDs (CD, Crohn’s disease-specific; CeD, celiac disease; IBD-shared, inflammatory bowel disease shared by Crohn’s disease and ulcerative colitis; JIA, juvenile idiopathic arthritis; PBC, primary biliary cirrhosis; PS, psoriasis; PsCh, primary sclerosing cholangitis; RA, rheumatoid arthritis; UC, ulcerative colitis-specific) in which a particular gene is located. We extracted the information about the function prediction from GeneNetwork [57] and listed the top 10 most significant Gene Ontology terms (GO) for biological process and another top 10 most significant GO terms for molecular function together with the *P*-value. GO terms containing ‘tumor necrosis factor (TNF) pathway’ or ‘T-cell/lymphocyte event’ in their description are highlighted in yellow. Additional file 19: Figure S10. Region IL21/IL21-AS1 as an example of a locus with a prioritized cell type. **(A)** Genomic overview of the region including four protein-coding genes (KIAA1109, ADAD1, IL2, IL21) and one lncRNA gene (IL21-AS1). **(B)** Expression of genes located in this region. T-helper 1 (Th1) cells are prioritized based on the co-expression levels of IL21 and IL21-AS1 (red ellipse).

## Abbreviations

AID: autoimmune and immune-related disorder
CD: Crohn’s disease
CeD: celiac disease
ENCODE: Encyclopedia of DNA Elements
eQTL: expression quantitative trait locus
GO: Gene Ontology
GWAS: genome-wide association studies
HLA: human leukocyte antigen
IBD: inflammatory bowel disease
IL: interleukin
JIA: juvenile idiopathic arthritis
lncRNA: long non-coding RNA
NK: natural killer
PBC: primary biliary cirrhosis
PBMC: peripheral blood mononuclear cell
PS: psoriasis
PsCh: primary sclerosing cholangitis
RA: rheumatoid arthritis
RPKM: reads per kilobase per million mapped reads
SNP: single-nucleotide polymorphism
Th: T-helper
Th0: primary T-helper
ThP: precursor T-helper
UC: ulcerative colitis
