# Supplementary figures and images for: Expression profiles of long non-coding RNAs located in autoimmune disease-associated regions reveal immune cell-type specificity
Source: Genome Med. 2014 Oct 28;6(10):88. doi: 10.1186/s13073-014-0088-0 (PMC4240855; doi:10.1186/s13073-014-0088-0)

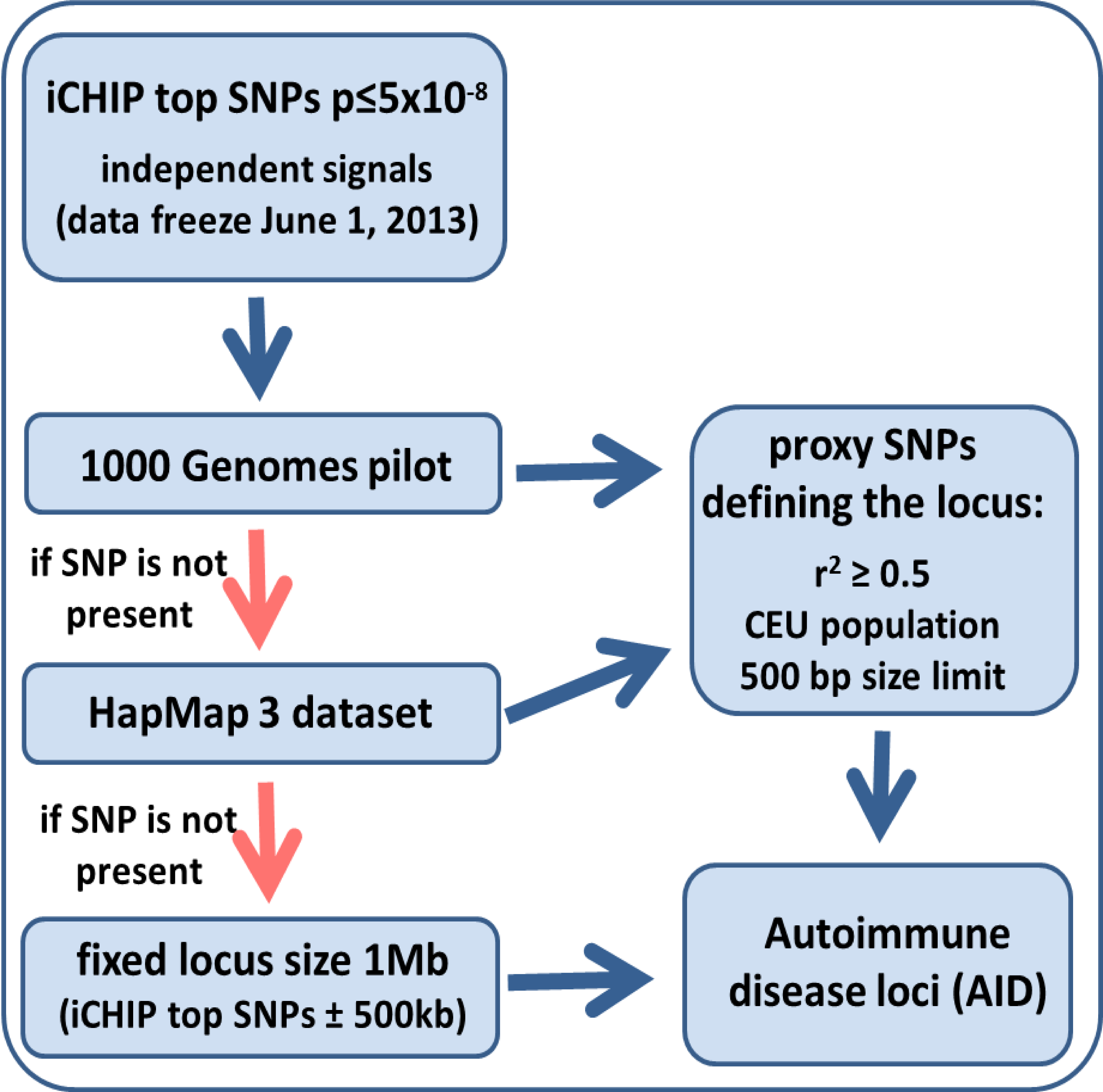

Supplement: Additional file 1: Figure S1. — A schematic illustration of our procedure to define autoimmune disease loci. The independent association signals in regions with multiple associations were defined using stepwise logistic regression conditioning on the most significant variant. Disease-associated loci were defined as regions containing the top SNP and its proxy SNPs (r2 ≥ 0.5) selected either from the 1000 Genomes Pilot (1000G) dataset or from the HapMap 3 dataset [32,33]. The disease locus was defined as a region with a fixed size of 1 Mb (top SNP ±500 kb) when the top SNP was absent from 1000G and HapMap3. We defined a disease locus as having a fixed 1 Mb size only in those cases that the disease-associated SNP was absent from the 1000 Genomes pilot and the HapMap 3 datasets. This was the case for only four AID loci: one shared by five AIDs (JIA, PBC, PS, RA, IBD-shared), one shared by RA and IBD; one shared by CeD and RA; and one CeD-only locus. [file 13073_2014_88_MOESM1_ESM.png]

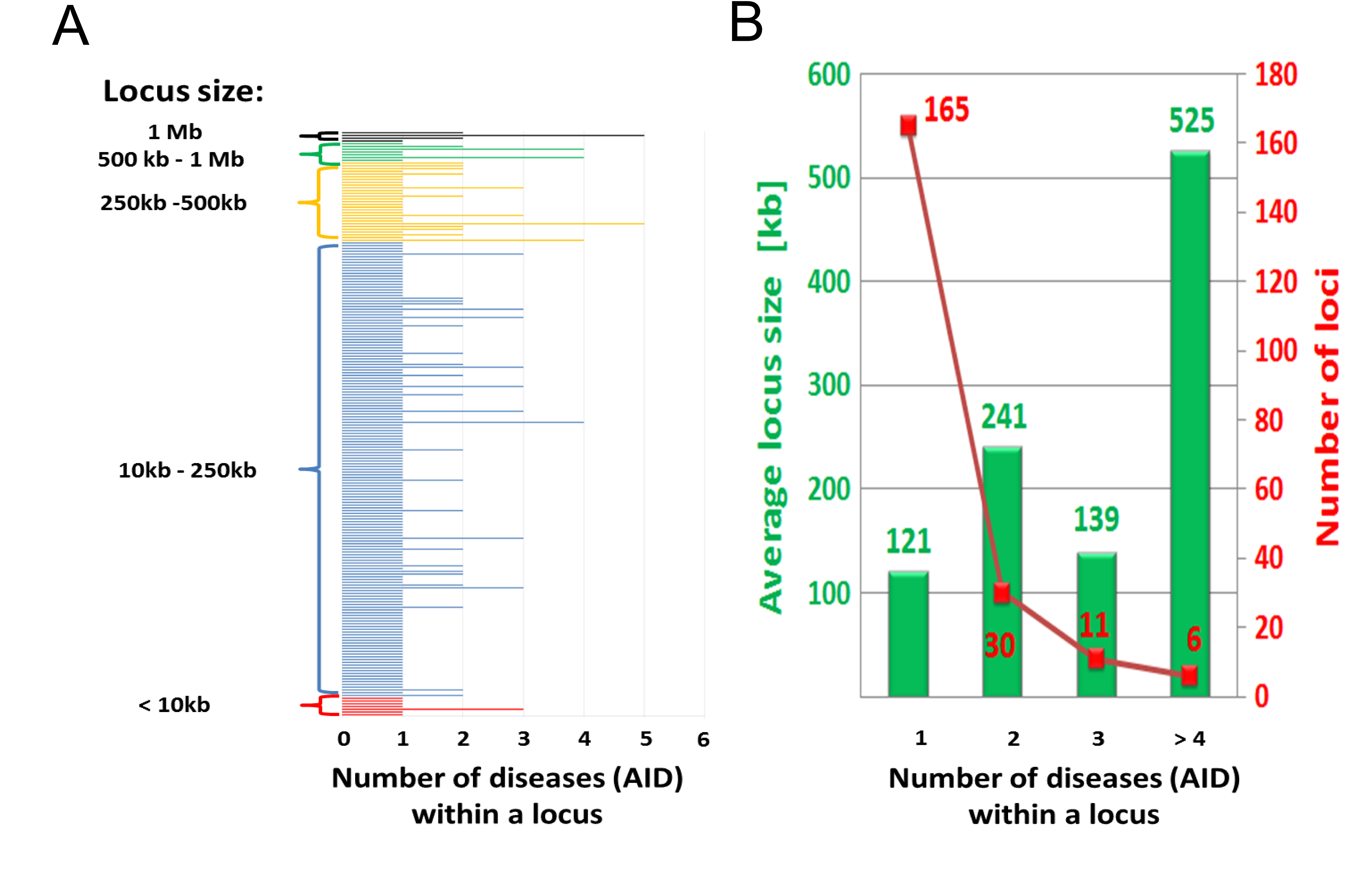

Supplement: Additional file 4: Figure S2. — Absence of a relationship between locus size and the number of autoimmune diseases (AIDs) associated with those loci. (A) Number of diseases sharing one AID locus (x-axis) versus locus size distribution (y-axis). All the AID loci were grouped in five differently colored segments based on the size range of each locus (<10 kb; 10 to 250 kb; 250 to 500 kb; 500 kb to 1 Mb; 1 Mb). (B) The characteristics and distribution of AID loci. The number of AIDs associated with a given locus is plotted on the x-axis. The green bars represent the average locus size in base pairs (kb) on the left-hand y-axis. The red line corresponds to the number of loci in each group of loci shared by a certain number of AIDs on the right-hand y-axis. [file 13073_2014_88_MOESM4_ESM.png]

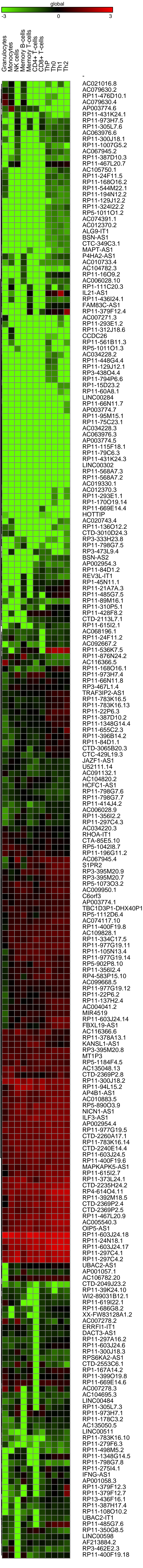

Supplement: Additional file 17: Figure S9. — RNA sequencing analysis of gene expression in seven peripheral blood leukocyte and four cord blood T-helper cell populations. Each panel is shown as a separate file with a higher resolution so that the gene IDs can be read easily. (A-D) The heat maps show expression of all genes located in AID loci ((A) lncRNAs, (B) protein-coding genes) and AID genes shared by at least two diseases ((C) lncRNAs, (D) protein-coding genes) in all 11 cell types (granulocytes, monocytes, NK cells, B cells, memory T cells (both CD4+ and CD8+), naive CD4+and naive CD8+ T cells (cytotoxic T cells), precursor T-helper cells (ThP), primary T-helper cells (Th0) and polarized T cells (Th1, Th2)). In the color scheme, saturated red indicates three-fold up-regulation, saturated green indicates three-fold down-regulation, and black indicates unchanged expression. (A) RNA sequencing analysis of gene expression in seven peripheral blood leukocyte and four cord blood T-helper cell populations - 240 AID lncRNAs. (B) RNA sequencing analysis of gene expression in seven peripheral blood leukocyte and four cord blood T-helper cell populations - 626 AID protein-coding genes. (C) RNA sequencing analysis of gene expression in seven peripheral blood leukocyte and four cord blood T-helper cell populations - 61 lncRNAs shared between at least two AIDs. (D) RNA sequencing analysis of gene expression in seven peripheral blood leukocyte and four cord blood T-helper cell populations - 186 protein-coding genes shared between at least two AIDs. [file 13073_2014_88_MOESM17_ESM.zip › 4701398771324073_add18.pdf]

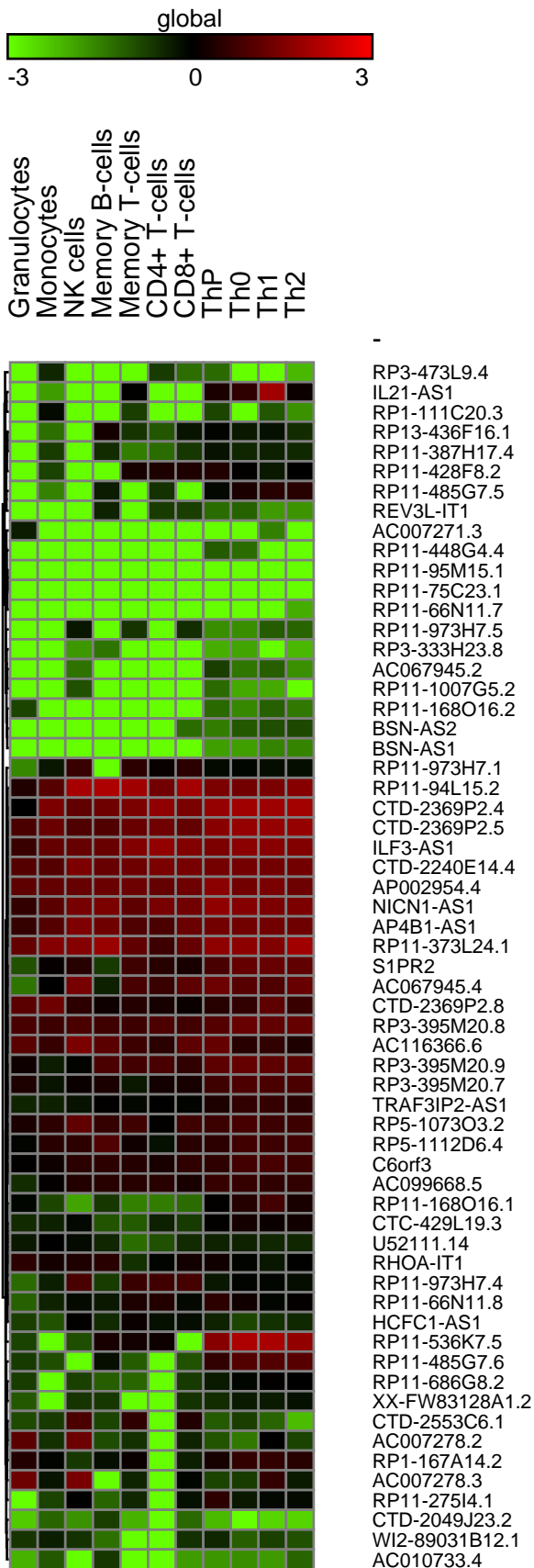

Supplement: Additional file 17: Figure S9. — RNA sequencing analysis of gene expression in seven peripheral blood leukocyte and four cord blood T-helper cell populations. Each panel is shown as a separate file with a higher resolution so that the gene IDs can be read easily. (A-D) The heat maps show expression of all genes located in AID loci ((A) lncRNAs, (B) protein-coding genes) and AID genes shared by at least two diseases ((C) lncRNAs, (D) protein-coding genes) in all 11 cell types (granulocytes, monocytes, NK cells, B cells, memory T cells (both CD4+ and CD8+), naive CD4+and naive CD8+ T cells (cytotoxic T cells), precursor T-helper cells (ThP), primary T-helper cells (Th0) and polarized T cells (Th1, Th2)). In the color scheme, saturated red indicates three-fold up-regulation, saturated green indicates three-fold down-regulation, and black indicates unchanged expression. (A) RNA sequencing analysis of gene expression in seven peripheral blood leukocyte and four cord blood T-helper cell populations - 240 AID lncRNAs. (B) RNA sequencing analysis of gene expression in seven peripheral blood leukocyte and four cord blood T-helper cell populations - 626 AID protein-coding genes. (C) RNA sequencing analysis of gene expression in seven peripheral blood leukocyte and four cord blood T-helper cell populations - 61 lncRNAs shared between at least two AIDs. (D) RNA sequencing analysis of gene expression in seven peripheral blood leukocyte and four cord blood T-helper cell populations - 186 protein-coding genes shared between at least two AIDs. [file 13073_2014_88_MOESM17_ESM.zip › 4701398771324073_add20.pdf]

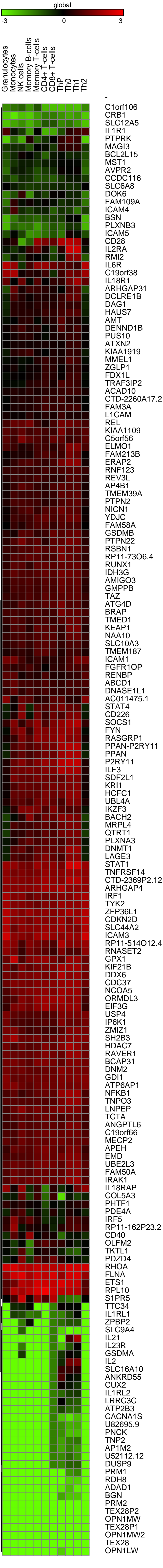

Supplement: Additional file 17: Figure S9. — RNA sequencing analysis of gene expression in seven peripheral blood leukocyte and four cord blood T-helper cell populations. Each panel is shown as a separate file with a higher resolution so that the gene IDs can be read easily. (A-D) The heat maps show expression of all genes located in AID loci ((A) lncRNAs, (B) protein-coding genes) and AID genes shared by at least two diseases ((C) lncRNAs, (D) protein-coding genes) in all 11 cell types (granulocytes, monocytes, NK cells, B cells, memory T cells (both CD4+ and CD8+), naive CD4+and naive CD8+ T cells (cytotoxic T cells), precursor T-helper cells (ThP), primary T-helper cells (Th0) and polarized T cells (Th1, Th2)). In the color scheme, saturated red indicates three-fold up-regulation, saturated green indicates three-fold down-regulation, and black indicates unchanged expression. (A) RNA sequencing analysis of gene expression in seven peripheral blood leukocyte and four cord blood T-helper cell populations - 240 AID lncRNAs. (B) RNA sequencing analysis of gene expression in seven peripheral blood leukocyte and four cord blood T-helper cell populations - 626 AID protein-coding genes. (C) RNA sequencing analysis of gene expression in seven peripheral blood leukocyte and four cord blood T-helper cell populations - 61 lncRNAs shared between at least two AIDs. (D) RNA sequencing analysis of gene expression in seven peripheral blood leukocyte and four cord blood T-helper cell populations - 186 protein-coding genes shared between at least two AIDs. [file 13073_2014_88_MOESM17_ESM.zip › 4701398771324073_add21.pdf]

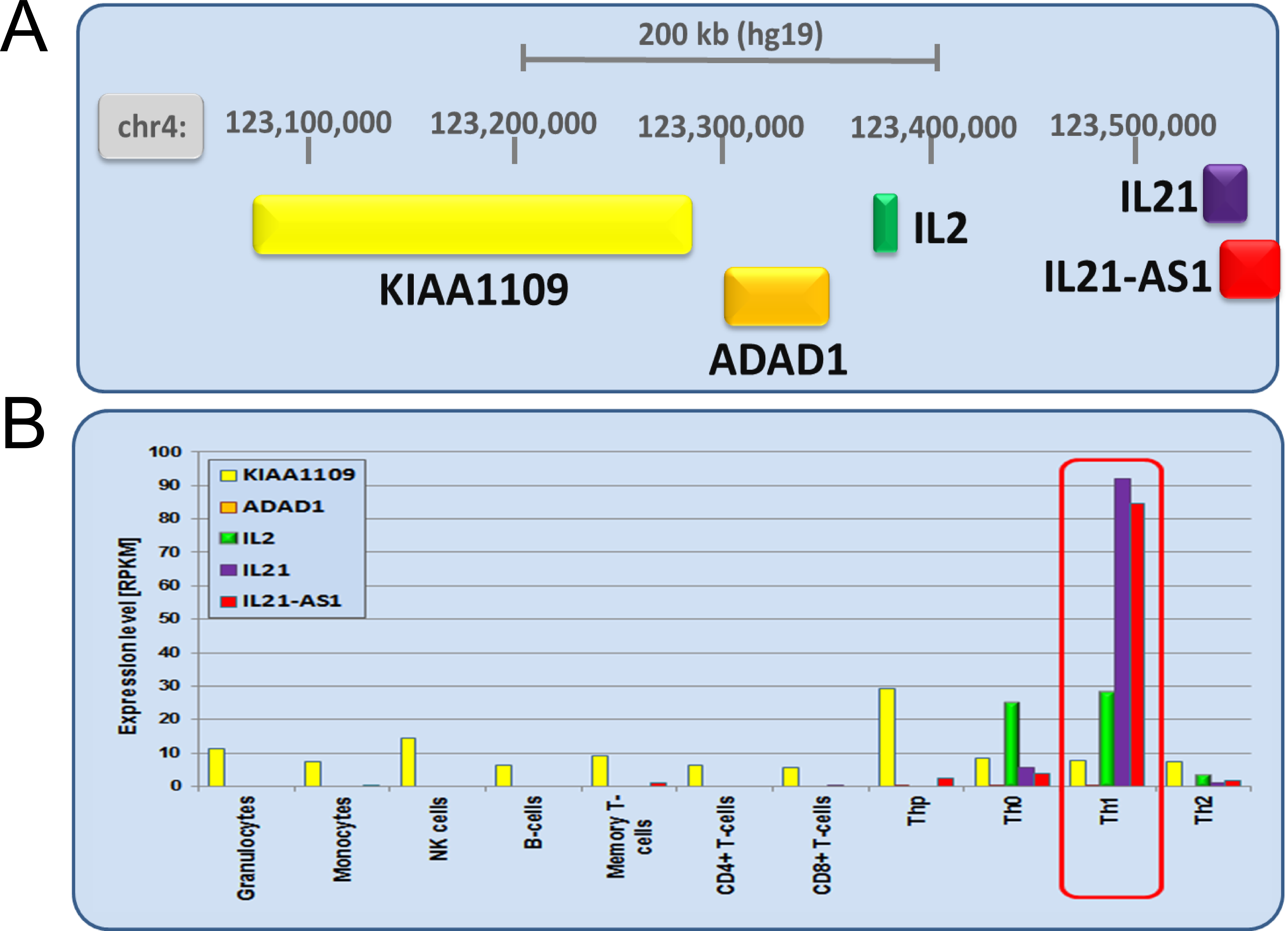

Supplement: Additional file 19: Figure S10. — Region IL21/IL21-AS1 as an example of a locus with a prioritized cell type. (A) Genomic overview of the region including four protein-coding genes (KIAA1109, ADAD1, IL2, IL21) and one lncRNA gene (IL21-AS1). (B) Expression of genes located in this region. T-helper 1 (Th1) cells are prioritized based on the co-expression levels of IL21 and IL21-AS1 (red ellipse). [file 13073_2014_88_MOESM19_ESM.png]
