# Supplementary material for: Expression profiles of long non-coding RNAs located in autoimmune disease-associated regions reveal immune cell-type specificity
Source: Genome Med. 2014 Oct 28;6(10):88. doi: 10.1186/s13073-014-0088-0 (PMC4240855; doi:10.1186/s13073-014-0088-0)

Figure S8

A) Crohn's disease-specific

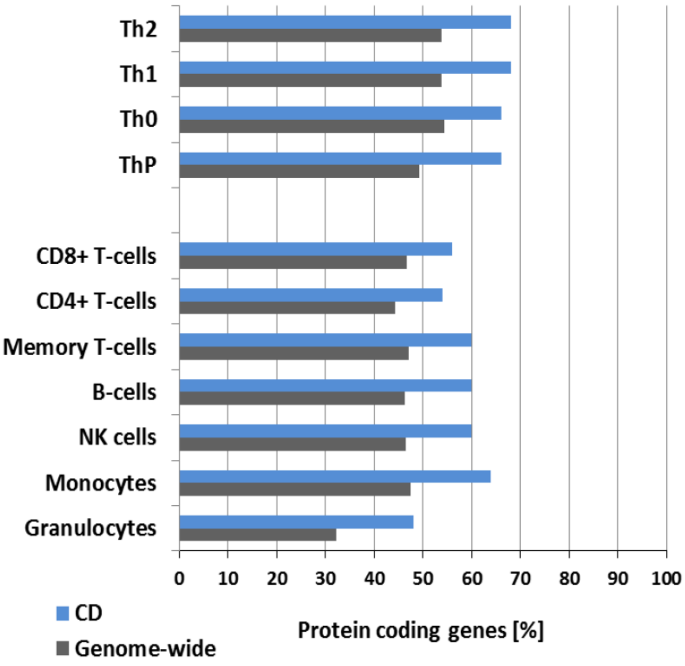

B) Celiac disease

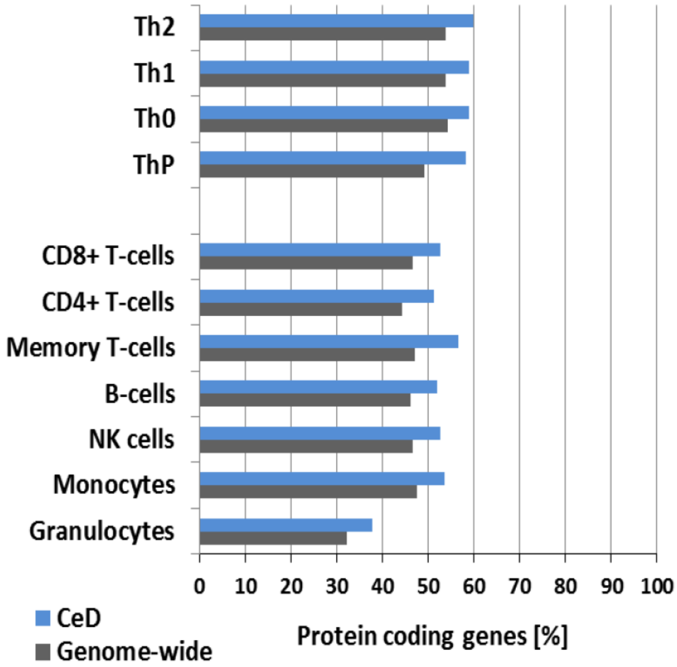

C) Inflammatory bowel disease-sh.

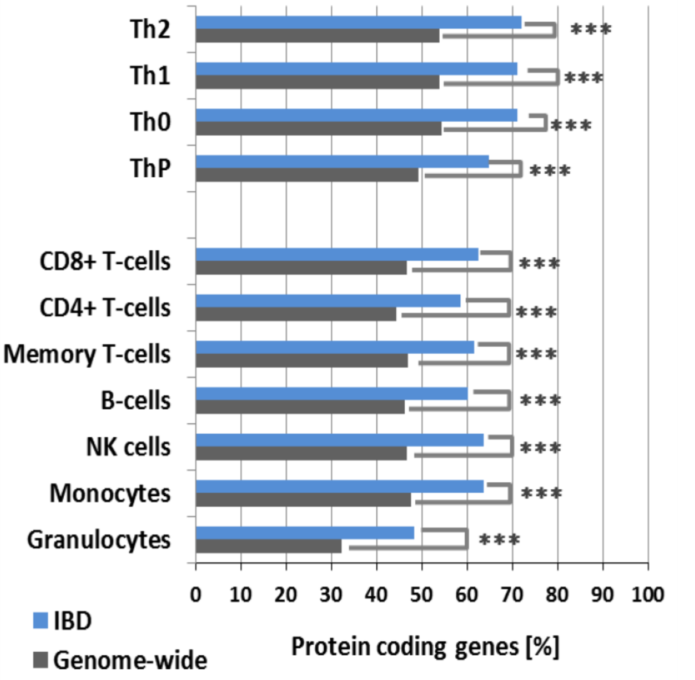

D) Juvenile idiopathic arthritis

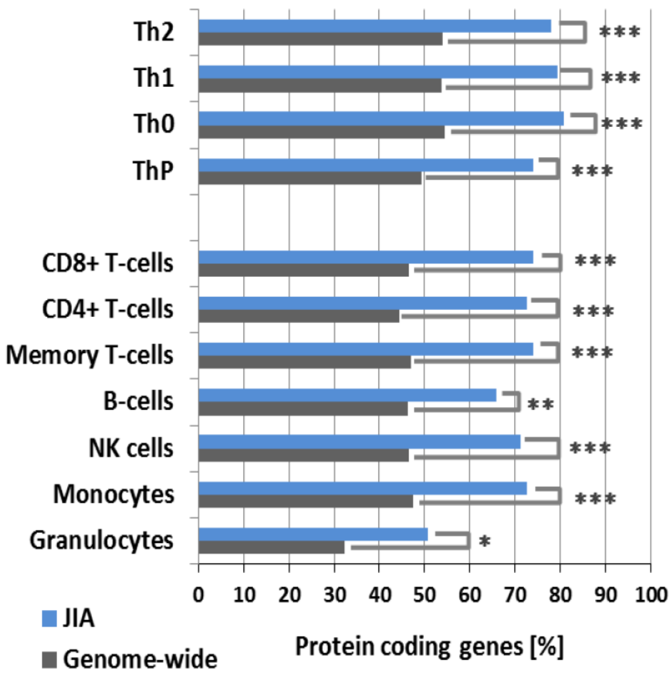

E) Primary biliary cirrhosis

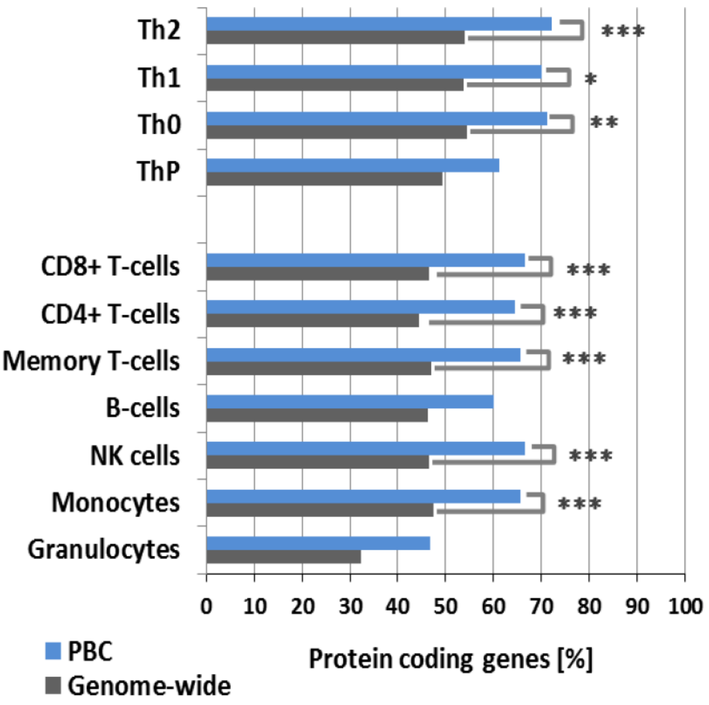

F) Psoriasis

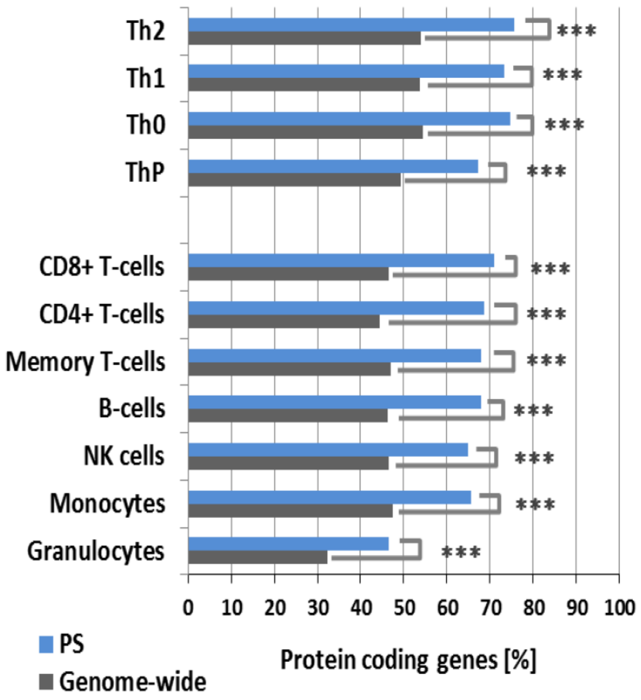

G) Primary sclerosing cholangitis

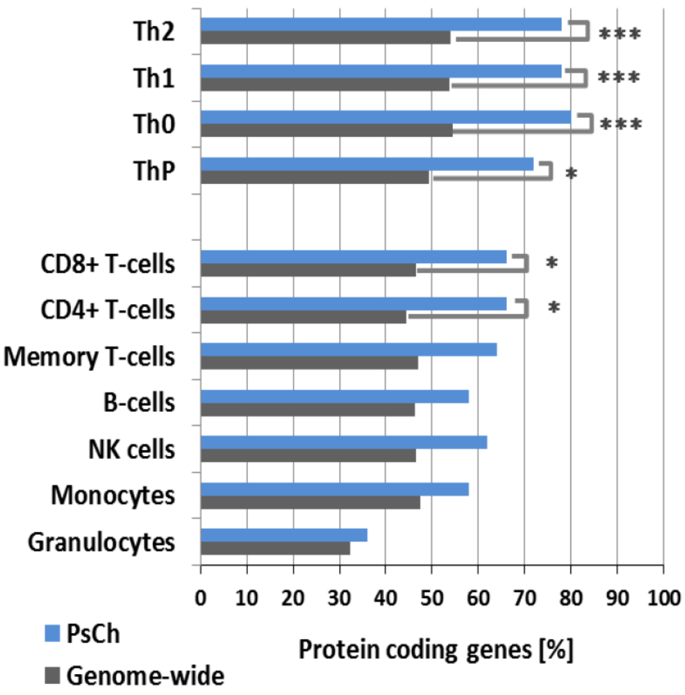

H) Rheumatoid arthritis

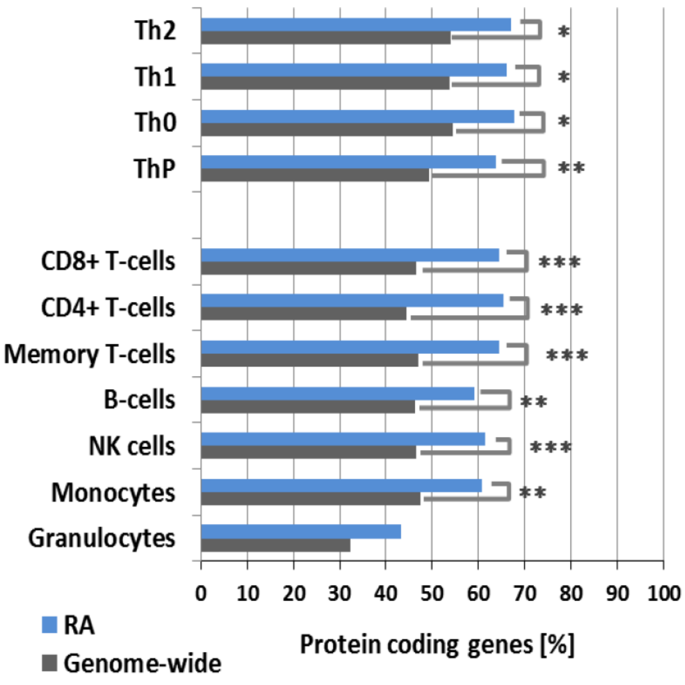

I) Ulcerative colitis-specific

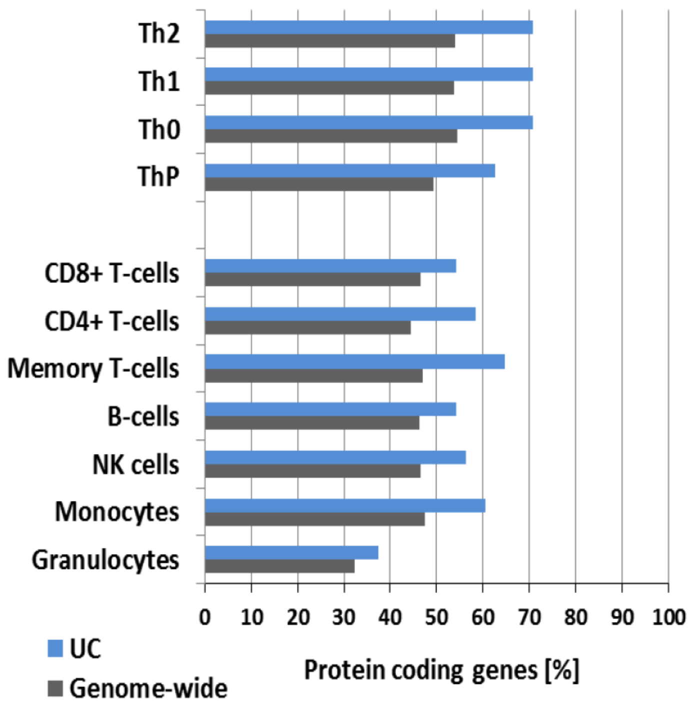

Supplement: Additional file 16: Figure S8. — Proportion of protein-coding genes expressed in seven peripheral blood leukocyte and four cord blood T-helper cell populations, per disease. For each disease, we compared the proportion of expressed coding genes (>2 RPKM) in the whole genome (genome-wide, gray) with the proportion of expressed disease-specific coding genes (red), and tested for differences using two-tailed Fisher’s exact test. Statistically significant enrichments (P-values) after Bonferroni correction for multiple testing are denoted by asterisks to show the different levels of significance (*α < 0.05; **α < 0.01; ***α < 0.005). (A) Crohn’s disease-specific; (B) celiac disease; (C) inflammatory bowel disease shared by Crohn’s disease and ulcerative colitis; (D) juvenile idiopathic arthritis; (E) primary biliary cirrhosis; (F) psoriasis; (G) primary sclerosing cholangitis; (H) rheumatoid arthritis; (I) ulcerative colitis-specific. [file 13073_2014_88_MOESM16_ESM.pdf]
