# Supplementary material for: Expression profiles of long non-coding RNAs located in autoimmune disease-associated regions reveal immune cell-type specificity
Source: Genome Med. 2014 Oct 28;6(10):88. doi: 10.1186/s13073-014-0088-0 (PMC4240855; doi:10.1186/s13073-014-0088-0)

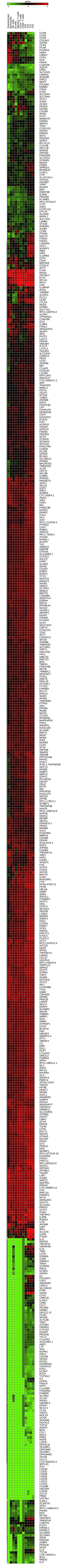

Supplement: Additional file 17: Figure S9. — RNA sequencing analysis of gene expression in seven peripheral blood leukocyte and four cord blood T-helper cell populations. Each panel is shown as a separate file with a higher resolution so that the gene IDs can be read easily. (A-D) The heat maps show expression of all genes located in AID loci ((A) lncRNAs, (B) protein-coding genes) and AID genes shared by at least two diseases ((C) lncRNAs, (D) protein-coding genes) in all 11 cell types (granulocytes, monocytes, NK cells, B cells, memory T cells (both CD4+ and CD8+), naive CD4+and naive CD8+ T cells (cytotoxic T cells), precursor T-helper cells (ThP), primary T-helper cells (Th0) and polarized T cells (Th1, Th2)). In the color scheme, saturated red indicates three-fold up-regulation, saturated green indicates three-fold down-regulation, and black indicates unchanged expression. (A) RNA sequencing analysis of gene expression in seven peripheral blood leukocyte and four cord blood T-helper cell populations - 240 AID lncRNAs. (B) RNA sequencing analysis of gene expression in seven peripheral blood leukocyte and four cord blood T-helper cell populations - 626 AID protein-coding genes. (C) RNA sequencing analysis of gene expression in seven peripheral blood leukocyte and four cord blood T-helper cell populations - 61 lncRNAs shared between at least two AIDs. (D) RNA sequencing analysis of gene expression in seven peripheral blood leukocyte and four cord blood T-helper cell populations - 186 protein-coding genes shared between at least two AIDs. [file 13073_2014_88_MOESM17_ESM.zip › 4701398771324073_add19.pdf]
